# Supplementary material for: Causal Relationship between Aging and Anorexia Nervosa: A White-Matter-Microstructure-Mediated Mendelian Randomization Analysis
Source: Biomedicines. 2024 Aug 16;12(8):1874. doi: 10.3390/biomedicines12081874 (PMC11351342; doi:10.3390/biomedicines12081874)
Supplement: Supplementary file 1 [file biomedicines-12-01874-s001.zip › Supplementary Figure/Figure S4/Singe SNP plot of MR analysis for TL to ubm-b-1807.pdf]

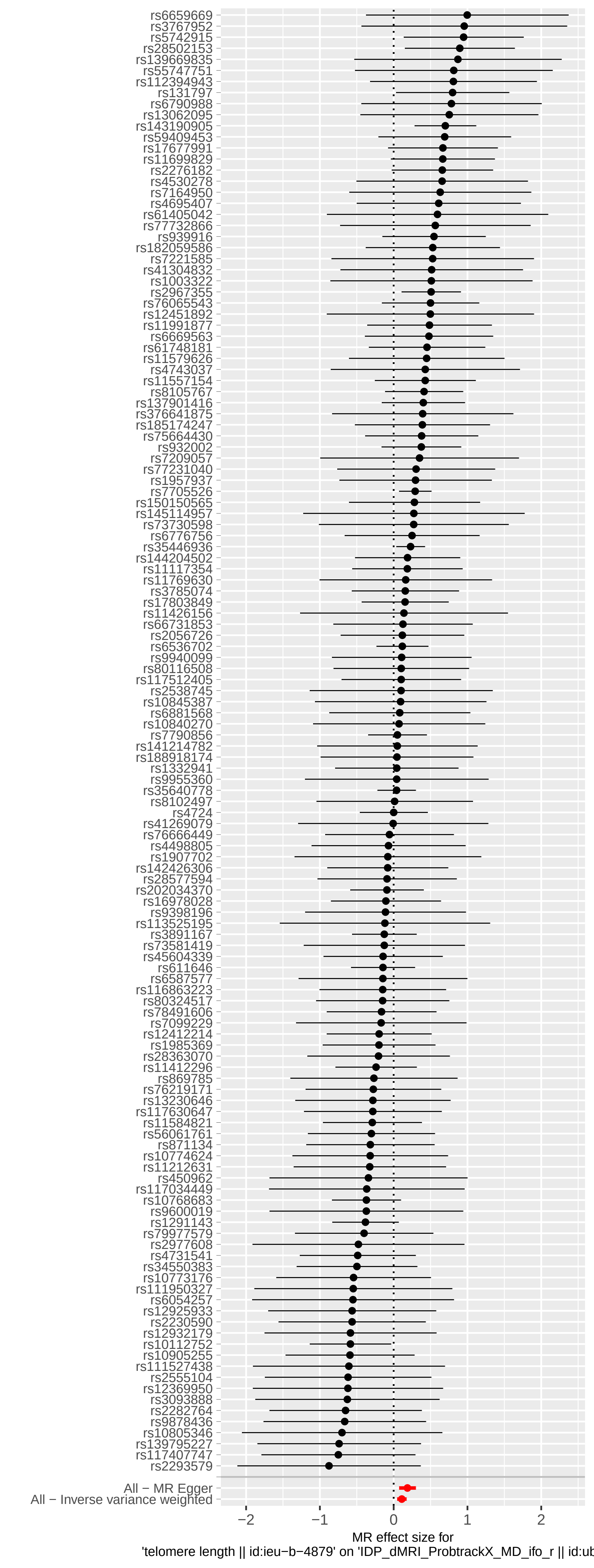
rs6659669
rs3767952
rs5742915
rs28502153
rs139669835
rs55747751
rs112394943
rs131797
rs6790988
rs13062095
rs143190905
rs59409453
rs17677991
rs11699829
rs2276182
rs4530278
rs7164950
rs4695407
rs61405042
rs77732866
rs939916
rs182059586
rs7221585
rs41304832
rs1003322
rs2967355
rs76065543
rs12451892
rs11991877
rs6669563
rs61748181
rs11579626
rs4743037
rs11557154
rs8105767
rs137901416
rs376641875
rs185174247
rs75664430
rs932002
rs7209057
rs77231040
rs1957937
rs7705526
rs150150565
rs145114957
rs73730598
rs6776756
rs35446936
rs144204502
rs11117354
rs11769630
rs3785074
rs17803849
rs11426156
rs66731853
rs2056726
rs6536702
rs9940099
rs80116508
rs117512405
rs2538745
rs10845387
rs6881568
rs10840270
rs7790856
rs141214782
rs188918174
rs1332941
rs9955360
rs35640778
rs8102497
rs4724
rs41269079
rs76666449
rs4498805
rs1907702
rs142426306
rs28577594
rs202034370
rs16978028
rs9398196
rs113525195
rs3891167
rs73581419
rs45604339
rs611646
rs6587577
rs116863223
rs80324517
rs78491606
rs7099229
rs12412214
rs1985369
rs28363070
rs11412296
rs869785
rs76219171
rs13230646
rs117630647
rs11584821
rs56061761
rs871134
rs10774624
rs11212631
rs450962
rs117034449
rs10768683
rs9600019
rs1291143
rs79977579
rs2977608
rs4731541
rs34550383
rs10773176
rs111950327
rs6054257
rs12925933
rs2230590
rs12932179
rs10112752
rs10905255
rs111527438
rs2555104
rs12369950
rs3093888
rs2282764
rs9878436
rs10805346
rs139795227
rs117407747
rs2293579
All − MR Egger
All − Inverse variance weighted
−2
−1
0
1
2
MR effect size for
'telomere length || id:ieu-b-4879' on 'IDP_dMRI_ProbtrackX_MD_ifo_r || id:ub
